# Ameliorating the Effect of Astragaloside IV on Learning and Memory Deficit after Chronic Cerebral Hypoperfusion in Rats

**DOI:** 10.3390/molecules20021904

**Published:** 2015-01-23

**Authors:** Sooyong Kim, Il-Hwan Kang, Jung-Bum Nam, Yoonchul Cho, Doo-Young Chung, Seung-Hwan Kim, Jeong-Soo Kim, Yong-Deok Cho, Eun-Ki Hong, Nak-Won Sohn, Jung-Won Shin

**Affiliations:** Department of East-West Medical Science, Graduate School of East-West Medical Science, Kyung Hee University, Yongin 446-701, Korea; E-Mails: kforreal@hanmail.net (S.K.); hukhihaha@hanmail.net (I.-H.K.); j789524@empal.com (J.-B.N.); phair@hanmail.net (Y.C.); ormd2020@naver.com (D.-Y.C.); hwany112@nate.com (S.-H.K.); kimjeongs@gmail.com (J.-S.K.); truthmam@naver.com (Y.-D.C.); ekhong09@hanmail.net (E.-K.H.); sohnnw@khu.ac.kr (N.-W.S.)

**Keywords:** astragaloside IV, cerebral hypoperfusion, memory impairment, neuronal apoptosis, oxidative damage, glial activation

## Abstract

Astragaloside IV (AS-IV) has been reported to have a prominent antioxidant effect and was proposed as a promising agent for the prevention of neurodegenerative disorders accompanied by cognitive impairment. The present study investigated the ameliorating effect of AS-IV on learning and memory deficits induced by chronic cerebral hypoperfusion in rats. Rats were treated with two doses of AS-IV (10 and 20 mg/kg, i.p.) daily for 28 days starting from the 5th week after permanent bilateral common carotid artery occlusion. AS-IV treatment (at dose of 20 mg/kg) significantly improved the spatial learning and memory deficits assessed using the Morris water maze test in rats with chronic cerebral hypoperfusion. AS-IV significantly attenuated neuronal apoptosis as well as the levels of superoxide dismutase and lipid peroxidation markers, including malondialdehyde and 4-hydroxy-2-nonenal, in the hippocampus. AS-IV also significantly reduced 8-hydroxy-2’-deoxyguanosine expression, a maker of oxidative DNA damage, while significantly inhibited the astrocyte and microglia activation in the hippocampus. The results indicate that AS-IV has therapeutic potential for the prevention of dementia caused by cerebral hypoperfusion and suggest that the ameliorating effect of AS-IV on learning and memory deficits might be the result of suppressing neuronal apoptosis and oxidative damage in the hippocampus.

## 1. Introduction

Cerebral hypoperfusion is associated with impaired cognitive functions both in Alzheimer’s disease (AD) and vascular dementia (VaD), which are the more common causes of cognitive impairment in aging. Patients with AD and subcortical ischemic VaD show marked reductions in cerebral blood flow (CBF) in the brain regions related to cognitive functions [1]. Chronic cerebrovascular hypoperfusion eventually results in neuronal damage [2] and is responsible for many pathophysiological changes in the brain, such as deranged metabolism, protein synthesis abnormalities, neuronal damage, glia activation, neurotransmission turnover and signaling, and white matter lesions [3]. Moreover, oxidative stress of vascular origin as a result of chronic cerebrovascular hypoperfusion plays a significant role in the onset and progression of neurodegenerative diseases [4]. During cerebral ischemia, reactive oxygen species (ROS) are generated in excess through the reduction of oxygen. ROS result in the lipid peroxidation and cleavage of DNA subsequently leading to neuronal damage. Significant elevations of malondialdehyde (MDA) and 4-hydroxy-2-nonenal (4-HNE), products of lipid peroxidation, and 8-hydroxy-2’-deoxyguanosine (8-OHdG), a maker of oxidative damage in intact DNA, have been reported in AD and VaD patients [5,6]. Oxidative stress and oxidative DNA damage are important factors involved in the pathogenesis of mixed AD and VaD [7]. These suggest that treating patients with antioxidants may prevent and delay the progression of the disease.

**Figure 1:**
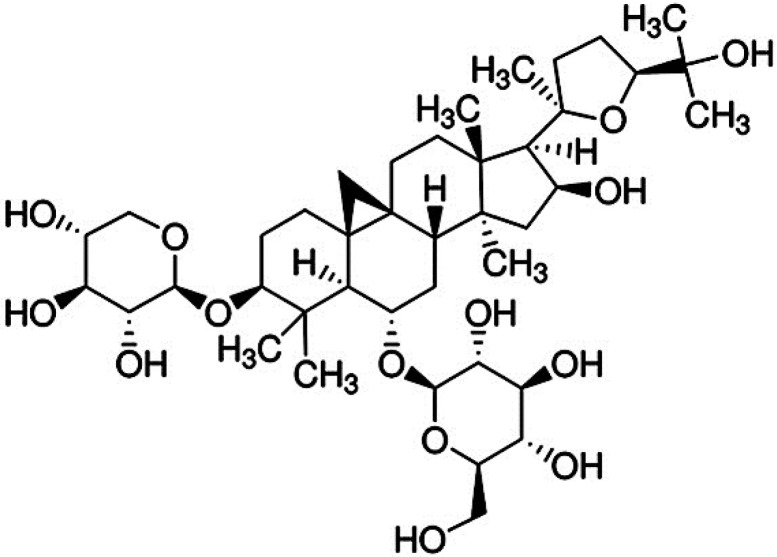
Chemical structure of astragaloside IV.

Astragaloside IV (AS-IV, Figure 1), a lanolin alcohol-shaped tetracyclic triterpenoid saponin is a major active component in Huangqi (*Astragali Radix*). It is used for the treatment of cardiovascular, hepatic, and renal disorders with a wide range of treatment effects [8]. Research has demonstrated that AS-IV has a prominent antioxidant effect. AS-IV inhibits the generation of ROS [9], reduces lipid peroxidation [10], and increases antioxidant enzymes [11]. AS-IV regulates nitric oxide and promotes angiogenesis through the JAK2/STAT3 and ERK1/2 pathways [12]. AS-IV exerts neuroprotective effects against ischemic brain injury by its antioxidant [13], anti-inflammatory [14], anti-apoptotic [15] and blood-brain barrier protective properties [16]. Recently, it was reported that AS-IV reduced ROS generation and neuronal apoptosis in an amyloid beta (Aβ) rich environment and was suggested that AS-IV may be a promising agent for the prevention and treatment of neurodegenerative disorders such as AD [17].

Under the hypothesis that the antioxidant and anti-apoptotic properties of AS-IV will exert influence on ameliorating learning and memory impairments, the present study investigated the effect of AS-IV in chronic cerebral hypoperfusion model of rats induced by permanent bilateral common carotid artery occlusion (pBCAO). In this study, we demonstrated that AS-IV improved learning and memory deficits via inhibiting oxidative damage, neuronal apoptosis, and glial activation in the hippocampus after chronic cerebral hypoperfusion.

## 2. Results and Discussion

### 2.1. Effects on Learning and Memory Impairment in the pBCAO Rats

A moderate but persistent reduction in regional CBF compromises memory processes and contributes to the development and progression of dementia [18]. The pBCAO in rats is the most common method to investigate the effects of chronic cerebral hypoperfusion on cognitive dysfunction and neurodegenerative processes. Surgical ligation of both common carotid arteries in rats produces a chronic and global cerebral hypoperfusion state [19]. After the pBCAO in rats, the CBF in the cerebral cortex and white matter areas was recorded to ~35%–45% of the control level, the CBF in the hippocampus decreasing to a lesser extent to ~60% of the control [20,21]. The CBF values started to gradually recover at one week, but remained significantly lower than the control values four weeks after the pBCAO induction [21]. It was also reported that the pBCAO rats demonstrated learning and memory impairments in Morris water maze tasks from four weeks up to 20 weeks after the ligation of the vessels [22]. Based on previous reports, a 4-week AS-IV treatment schedule was chosen for the present study, staring from the 5th week after the onset of pBCAO upto the 9th week after which the Morris water maze tasks were performed.

**Figure 2:**
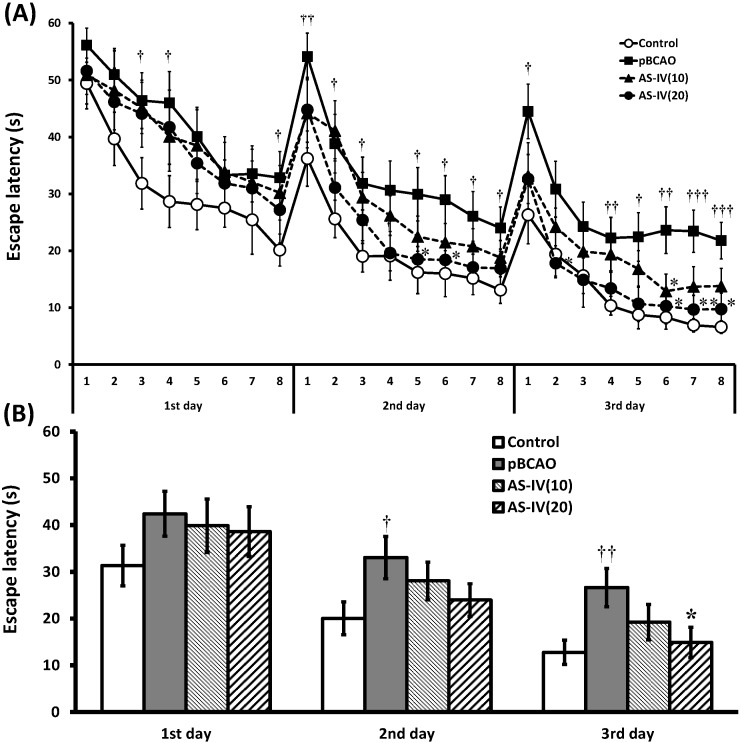
Effect of astragaloside IV (AS-IV) on the acquisition training trials. The escape latency of total trials for 3 days was significantly different between the control, pBCAO, AS-IV(10), and AS-IV(20) groups (**A**). AS-IV showed significantly shorter escape latency on the 3rd day, particularly at a dose of 20 mg/kg, compared to the pBCAO group (**B**). Data are presented as mean ± SEM (*n* = 12 in each group; **†**, *p* < 0.05; **††**, *p* < 0.01; **†††**, *p* < 0.001 between the control and the pBCAO groups; *, *p* < 0.05 between the pBCAO and AS-IV groups).

In the present study, the escape latencies for the 24 trials done during the three days of acquisition training showed a significant difference among the study groups (F_3,92_ = 5.081, *p* = 0.003). The escape latency of the pBCAO group was significantly longer than the control group (F_1,46_ = 16.474, *p* = 0.0002). The AS-IV(10) group did not show comparable results in the escape latency compared to the pBCAO group (F_1,46_ = 2.392, *p* = 0.129), while the AS-IV(20) group demonstrated significantly shorter escape latency compared to that of the pBCAO group (F_1,46_ = 5.711, *p* = 0.021) (Figure 2A). The average escape latency on the 1st day was similar among the study groups. On the 2nd day, the average escape latency of the pBCAO group showed significantly longer than that of the control group (*p* < 0.05), but those of AS-IV groups were not different compared to the pBCAO group. On the 3rd day, the pBCAO group also showed significantly longer escape latency than that of the control group (*p* < 0.01), while the AS-IV 20 mg/kg treatment significantly shorten the escape latency compared to that of the pBCAO group (*p* < 0.05) (Figure 2B).

In the retention trial done on the 4th day of the Morris water maze test, the swimming time spent in the various zones, number of target heading, time for the 1st target heading and memory score were used to estimate spatial memory. The pBCAO rats, including AS-IV treated animals, showed a small reduction (~5%) in swimming distances for 60 min compared to that of the control group, but the average swimming distances and swimming speeds in the retention test trials were similar among all study groups.

**Figure 3:**
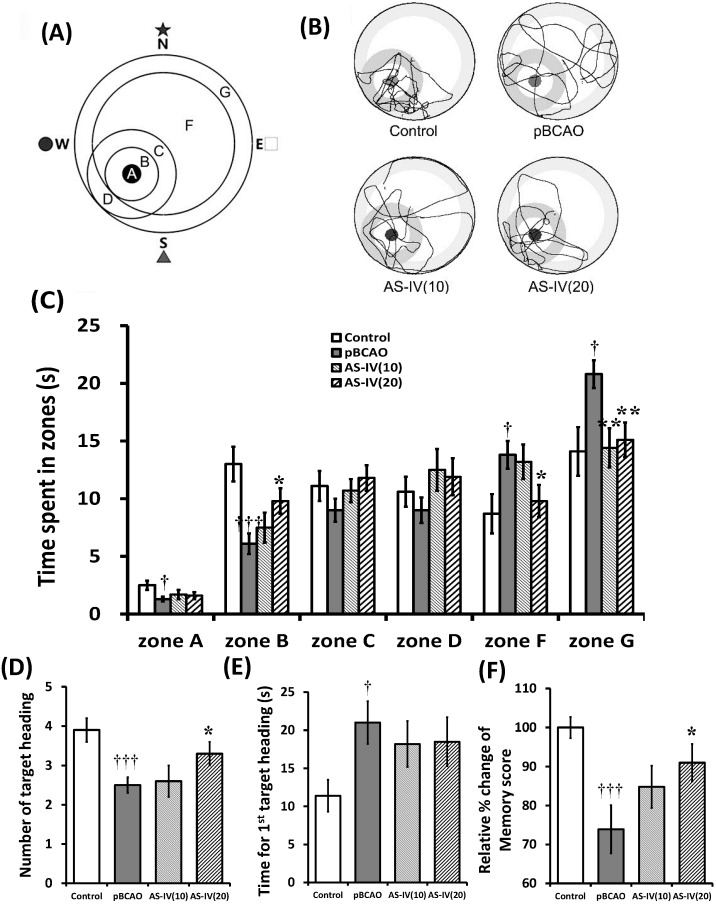
Effects of astragaloside IV (AS-IV) on the retention test. Computerized grid design which used in the retention test (**A**). Discrete zones are labeled with letters, zone A representing the platform site. Representative swimming tracts in study groups (**B**). AS-IV on the swimming time spent in discrete zones (**C**). AS-IV significantly prolonged the swimming time spent in zone B and significantly shortened it in zones F and G at a dose of 20 mg/kg. AS-IV significantly increased the number of target heading on platform site (**D**) and memory score (**F**) in the retention test at 20 mg/kg administration. The time for 1st target heading was not different (**E**). Data are presented as mean ± SEM (*n* = 12 in each group; **†**, *p* < 0.05; **†††**, *p* < 0.001 between the control and the pBCAO groups; *, *p* < 0.05; **, *p* < 0.01 between the pBCAO and AS-IV groups).

The pBCAO group spent significantly less time in zone A (the target platform site; *p* < 0.05) and zone B (*p* < 0.001), while they spent significantly longer time in zones F and G (*p* < 0.05, *p* < 0.01, respectively) compared to those of the control group. The AS-IV(10) group showed prolonged swimming time only in zone G (*p* < 0.01) compared to the pBCAO group. The AS-IV(20) group swam for a significantly longer time in zone B (*p* < 0.05) and significantly shorter time in zone F (*p* < 0.05) and zone G (*p* < 0.01) compared to the pBCAO group (Figure 3A,B). The pBCAO group had significantly less number of target heading (*p* < 0.001), had a significantly longer time for the 1st target heading (*p* < 0.05), and had significantly decreased memory score (*p* < 0.01) compared to those of the control group (Figure 3C–E). The AS-IV(10) group did not differ with the pBCAO group in all indicators measured.

The AS-IV(20) group showed significant increase in the number of target heading and memory score (*p* < 0.05, respectively) compared to those of the pBCAO group (Figure 3C–E). In previous reports, extract of Astragalus (20 and 40 mg/kg treatment for 21 days) and astragalosides (16 and 32 mg/kg treatment for 14 days) improved learning and memory impairments induced by Aβ_(25-35)_ and dexamethasone treatment in rats [23,24]. The present study shows AS-IV’s ameliorating effect (20 mg/kg treatment for 28 days) on spatial learning and memory deficits in chronic cerebral hypoperfusion induced by pBCAO in rats.

### 2.2. Effects on Neuronal Apoptosis in the Hippocampus of the pBCAO Rats

The spatial learning task of the pBCAO rats was impaired even after the CBF has returned to baseline, more than six months after the onset of pBCAO [18,25]. From these observations, it seems that the memory deficit correlated with the pBCAO-induced permanent neuronal damage rather than the cerebral hypoperfusion itself. 

**Figure 4:**
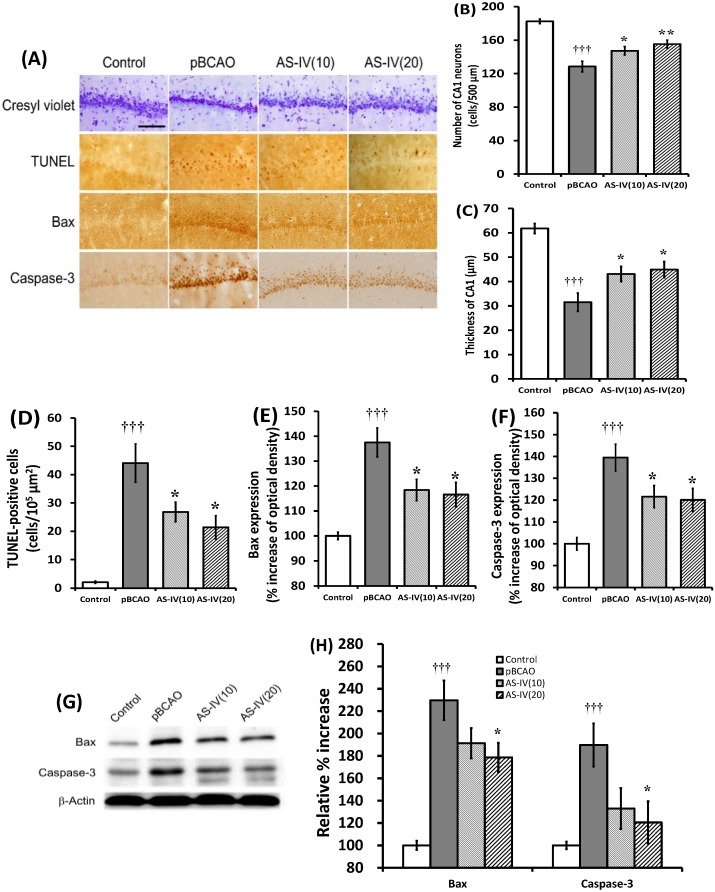
Effects of astragaloside IV (AS-IV) on neuronal apoptosis in the hippocampus. Representative photographs show the CA1 of the hippocampus stained with cresyl violet, TUNEL, Bax and caspase-3 expression (**A**). Scale bar is 50 µm, applicable to all sections. AS-IV, at both 10 and 20 mg/kg dose, significantly attenuated the neuronal loss and thinned thickness of the CA1 (**B**,**C**). AS-IV also significantly reduced TUNEL-positive cells (**D**) and significantly attenuated the upregulation of Bax (**E**) and caspase-3 (**F**) in the CA1 of the hippocampus at both 10 and 20 mg/kg treatment. Representative western blots illustrating differences in the bands of Bax and caspase-3 (**G**). The upregulation of Bax and caspase-3 protein expression was significantly attenuated by AS-IV at 20 mg/kg dose (**H**). Data are presented as mean ± SEM (*n* = 6 in each group; **†††**, *p* < 0.001 between the control and the pBCAO groups; *, *p* < 0.05; **, *p* < 0.01 between the pBCAO and AS-IV groups).

Neuronal death in the cerebral cortex and hippocampal CA1 region after chronic cerebral hypoperfusion directly causes progressive memory impairment. Previous studies reported that obvious loss of hippocampal neurons was not seen during the first week after the pBCAO induction [26]. At two weeks, 6%–29% of the animals exhibited hippocampal injury in the CA1 region. At four weeks, this had increased to 55%, while at 8–13 weeks, total hippocampal damage was observed in 67% of the pBCAO rats [19,26]. TUNEL-labeled apoptotic cell death was also detected in the CA1 and CA3 regions from two weeks after the onset of pBCAO [2]. In the present study, the eight weeks of chronic cerebral hypoperfusion by pBCAO induced significant reduction in the number of CA1 neurons and thickness of CA1 in the hippocampus (*p* < 0.001, respectively) compared to the control group (Figure 4A–C). Both 10 mg/kg (*p* < 0.05, respectively) and 20 mg/kg (*p* < 0.05 and *p* <0.01) AS-IV treatment doses significantly attenuated the loss of CA1 neurons and the reduction of CA1 thickness compared to those of the pBCAO group (Figure 4A–C). TUNEL-positive cells in the CA1 were markedly increased in the pBCAO group (*p* < 0.001), while AS-IV treatment attenuated it significantly at both 10 and 20 mg/kg treatment dose (*p* < 0.05, respectively) compared to the pBCAO group (Figure 4A,D). Bax and caspase-3 expression in the CA1 of hippocampus were markedly upregulated by pBCAO compared to the control group (*p* < 0.001, respectively). AS-IV treatment significantly attenuated the upregulation of Bax and caspase-3 expression at both 10 and 20 mg/kg treatment dose (*p* < 0.05, respectively) compared to the pBCAO group (Figure 4A,E,F). In quantitative evaluation using Western blot, the upregulation of Bax and caspase-3 expression in the hippocampal tissue was also significantly (*p* < 0.05) attenuated by AS-IV treatment at 20 mg/kg dose (Figure 4G,H). Previous reports demonstrated that AS-IV also attenuated neuronal apoptosis in focal cerebral ischemia [15], in subarachnoid hemorrhage [27] and in Aβ rich environment [17]. Based on previous reports and the present study, these results suggested that AS-IV had a protective effect against neuronal apoptosis in the hippocampus via suppressing the upregulation of Bax and caspase-3 expression. 10 mg/kg dose of AS-IV showed significant effect against neuronal loss and the upregulation of apoptosis-related molecules in the immunohistochemstry. However, that dose of AS-IV was not effective on learning or memory impairments. It might be due to the limitations of image analysis (measuring of gray levels, selection of interesting area, number of sections, *etc*.). In quantitative evaluation using western blot, the upregulation of Bax and caspase-3 expression in the hippocampal tissue was significantly attenuated by AS-IV treatment at 20 mg/kg dose.

### 2.3. Effects on Oxidative Brain Damage in the pBCAO Rats

**Figure 5:**
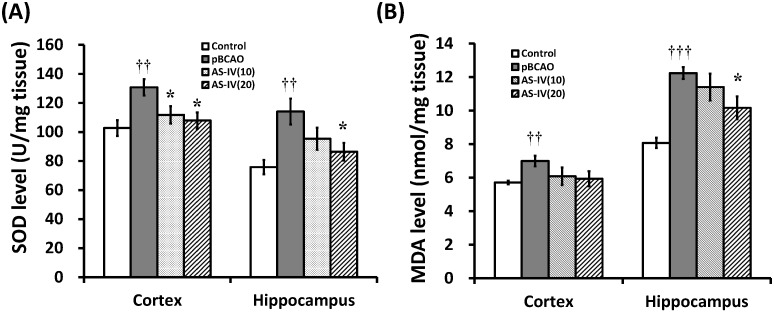
Effects of astragaloside IV (AS-IV) on the levels of SOD and MDA in brain tissue. AS-IV significantly reduced SOD levels in the cerebral cortex and the hippocampus at a dose of 20 mg/kg (**A**). AS-IV significantly attenuated the upregulation of MDA levels in the hippocampus at 20 mg/kg treatment (**B**). Data are presented as mean ± SEM (*n* = 6 in each group; **††**, *p* < 0.01; **†††**, *p* < 0.001 between the control and the pBCAO groups; *, *p* < 0.05 between the pBCAO and AS-IV groups).

Oxidative stress during cerebral hypoperfusion plays a key role in neuronal death. The brain is more vulnerable to oxidative damage due to its high metabolic rate, high polyunsaturated lipid content, and lower levels of endogenous antioxidant activity [3]. The excessive generation of ROS leads to oxidative damage to cellular proteins, lipids, and DNA. Superoxide dismutase (SOD) is one of the enzymatic defense mechanisms against free radical-induced oxidative stress [7]. In the present study, SOD levels of the pBCAO group were significantly increased in the cerebral cortex and hippocampal tissue (*p* < 0.01, respectively) compared to that of the control group. This may indicate a compensatory rise in antioxidant activity in response to the increased free radical generation. AS-IV treatment significantly reduced the SOD levels in the cerebral cortex at both 10 and 20 mg/kg treatment dose (*p* < 0.05, respectively) and in the hippocampal tissue at 20 mg/kg (*p* < 0.05) compared to that of the pBCAO group (Figure 5A). AS-IV treatment had significant reversing effect on the abnormal SOD levels in the two brain regions. This suggests that AS-IV may have attenuated the excessive generation of ROS and is consistent with the previous report that AS-IV has a significant antioxidant activity [9,10,11]. Excessive generation of ROS results in lipid peroxidation and subsequently gives rise to a number of secondary products. MDA is the principal and most studied product of polyunsaturated fatty acid peroxidation and should be considered as a marker of lipid peroxidation [28]. MDA levels of the pBCAO group were significantly increased in the cerebral cortex and hippocampal tissue (*p* < 0.01 and *p* < 0.001) compared to that of the control group. AS-IV treatment significantly attenuated MDA level in hippocampal tissue at 20 mg/kg (*p* < 0.05) compared to that of the pBCAO group (Figure 5B).

Peroxidative degradation of lipids also produces the lipid aldehyde 4-HNE [29]. Uncontrolled production of the lipid aldehyde leads to protein modification and initiation of a disease process. 4-HNE-derived epitopes have been detected in a variety of animal models of oxidative stress. Therefore, immunodetection of 4-HNE adducted proteins in tissue sections is used to assess lipid peroxidation [30]. Cellular and mitochondrial DNA in aerobic organisms are constantly being damaged through endogenous sources such as DNA instability, numerous ROS, and products of lipid peroxidation [31].

**Figure 6:**
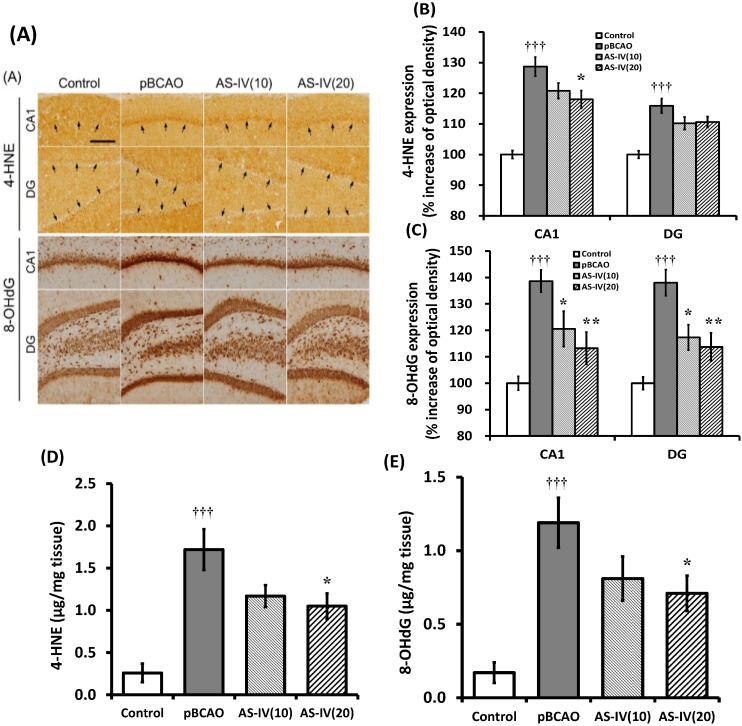
Effects of astragaloside IV (AS-IV) on 4-HNE and 8-OHdG expressions in hippocampal tissue. Representative photographs show the CA1 and DG regions of the hippocampus immuno-stained against 4-HNE and 8-OHdG (**A**). Scale bar is 100 µm, applicable to all sections. AS-IV at 20 mg/kg significantly attenuated the upregulation of 4-HNE expression in the CA1 of the hippocampus (**B**). AS-IV 10 and 20 mg/kg treatment significantly attenuated the upregulation of 8-OHdG expression in the CA1 and DG regions of the hippocampus (**C**). AS-IV treatment at 20 mg/kg significantly attenuated the upregulation of 4-HNE and 8-OHdG protein levels as determined by ELISA method (**D**,**E**). Data are presented as mean ± SEM (*n* = 6 in each group; **†††**, *p* < 0.001 between the control and the pBCAO groups; *, *p* < 0.05; **, *p* < 0.01 between the pBCAO and AS-IV groups).

The hydroxyl radical attacks DNA strands when it is produced adjacent to cellular and mitochondrial DNA causing the addition of DNA bases new radicals, which lead to the generation of a variety of oxidation products [32]. The biomarker 8-OHdG, an oxidized form of deoxyguanosine, is a commonly measured and sensitive marker of DNA damage [31]. Brain levels of 8-OHdG are increased in aging [33] and in animal models and patients with AD [34,35]. In the present study, lipid peroxidation and oxidative DNA damage were assessed by immunohistochemistry against anti-4-HNE and anti-8-OHdG antibodies. Chronic cerebral hypoperfusion produced significant upregulation of 4-HNE and 8-OHdG expression in the CA1 and DG regions of the hippocampus compared to the control group (Figure 6A). AS-IV treatment at 20 mg/kg significantly attenuated the 4-HNE upregulation only in the CA1 of the hippocampus (*p* < 0.05) compared to that of the pBCAO group (Figure 6A,B). AS-IV treatment significantly attenuated the 8-OHdG upregulation in the CA1 and DG region of the hippocampus both at 10 mg/kg (*p* < 0.05, respectively) and 20 mg/kg (*p* < 0.01, respectively) compared to that of the pBCAO group (Figure 6A,C). AS-IV treatment at 20 mg/kg also significantly (*p* < 0.05) attenuated the upregulation of 4-HNE and 8-OHdG protein levels in the hippocampal tissue as determined by ELISA method (Figure 6D,E). The results demonstrate that treatment with AS-IV (20 mg/kg) was effective in reducing oxidative brain damage induced by chronic cerebral hypoperfusion in the pBCAO rats. The results indicate that AS-IV treatment significantly reduced free radical damage to cells, enhanced antioxidant abilities, and attenuated lipid peroxidation products and oxidative DNA damage in the cerebral cortex and hippocampus caused by chronic cerebral hypoperfusion.

### 2.4. Effects on Glial Activation in the Hippocampus of the pBCAO Rats

**Figure 7:**
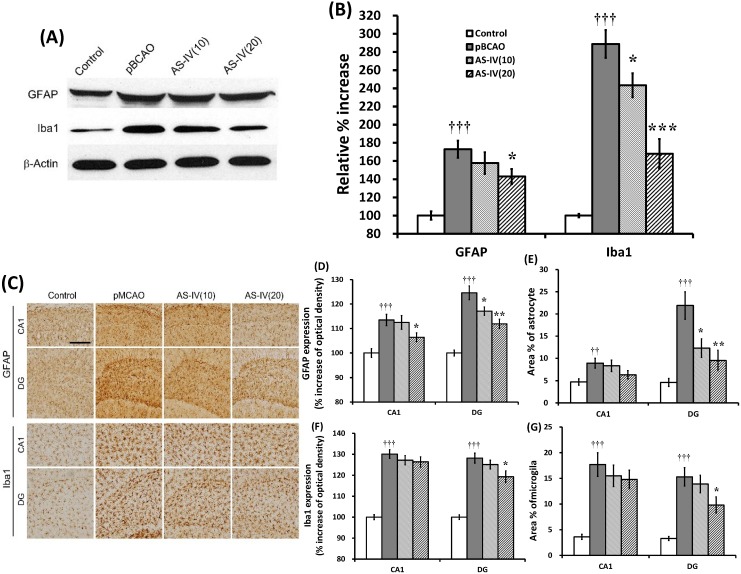
Effects of astragaloside IV (AS-IV) on GFAP and Iba1 expressions in hippocampal tissue. Representative western blots illustrating differences in the bands of GFAP and Iba1 (**A**). GFAP expression was significantly attenuated by AS-IV 20 mg/kg treatment, while Iba1 expression was significantly attenuated at both 10 and 20 mg/kg AS-IV treatment (**B**). Representative photographs show the CA1 and DG regions of the hippocampus immuno-stained against GFAP and Iba1 (**C**). Scale bar is 100 µm, applicable to all sections. AS-IV significantly attenuated the upregulation of GFAP expression in the CA1 and DG regions of the hippocampus (**D**) and significantly reduced the % area of the GFAP-labeled astrocytes in the DG region of the hippocampus (**E**) at both 10 and 20 mg/kg treatment. AS-IV at 20 mg/kg significantly attenuated the upregulation of Iba1 expression in the DG region of the hippocampus (**F**) and significantly reduced the % area of the Iba1-labeled microglia in the DG region of the hippocampus (**G**). Data are presented as mean ± SEM (*n* = 6 in each group; **††**, *p* < 0.01; **†††**, *p* < 0.001 between the control and the pBCAO groups; *, *p* < 0.05; **, *p* < 0.01; ***, *p* < 0.001 between the pBCAO and AS-IV groups).

The present study examined the effect of AS-IV on glial activation in the hippocampus affected by chronic cerebral hypoperfusion. The excessive and persistent glial activation is a characteristic feature of neuroinflammation and is considered a mediator for secondary damage, neuronal death and progression of disease [36]. Astrocytes and microglia are activated after cerebral hypoperfusion. Glial activation was already present at seven days and continued for up to three months after the pBCAO [37]. In the present study, the pBCAO group showed significant increase of GFAP (a marker of astrocyte activation) and Iba1 (a marker of microglial activation) protein expression in the hippocampal tissue (*p* < 0.001, respectively) compared to the control group (Figure 7A,B). AS-IV treatment significantly reduced GFAP protein expression at 20 mg/kg dose (*p* < 0.05) and Iba1 protein expression at doses of 10 and 20 mg/kg (*p* < 0.05 and *p* <0.001) compared to that of the pBCAO group (Figure 7A,B). Immunohistochemistry against GFAP and Iba1 showed significant activation of astrocytes and microglia in the CA1 and DG regions of the hippocampus of pBCAO rats compared to the control group (Figure 7C). Although 10 mg/kg of AS-IV treatment did not sufficiently attenuate the activations of astrocytes and microglia in the hippocampus (Figure 7C–G), the 20 mg/kg of AS-IV treatment significantly attenuated the expression density and the % areas of GFAP-expressing astrocytes (*p* < 0.01, respectively) and Iba1-expressing microglia (*p* < 0.05, respectively) in the DG region of the hippocampus compared to that of the pBCAO group (Figure 7C–G). The result suggests that AS-IV may be of benefit due to its ability to attenuate neuroinflammation which contribute to the progression of neurodegenerative diseases such as AD.

In many studies, a direct correlation among oxidative damage, neuronal apoptosis, and memory deficit under chronic cerebral hypoperfusion has been demonstrated [2,3,4,5]. AS-IV exerts antioxidant properties, including increasing SOD activity and reducing ROS generation and lipid peroxidation [9,10,11], consequentially protecting against ischemic brain injury [13,14,16]. AS-IV reduces neuronal apoptosis through inhibiting c-jun N-terminal kinase 3 expression and Bax-mediated pathway after cerebral ischemia [9,15]. AS-IV attenuates Aβ neurotoxicity via inhibiting the mitochondrial permeability transition pore opening and ROS generation [17]. Astragaloside improves cognitive impairment via reducing amyloid precursor protein expression [24]. These actions may help explain how AS-IV ameliorate cognitive impairment after cerebral hypoperfusion. In our present study, there is some shortages to explain the action mechanism of AS-IV on ameliorating cognitive impairment. We need more detailed tests to the efficacy of administration period, comparison with other well-known and studied antioxidant drug, and *in vitro* study elucidating molecular mechanisms. 

In the present study, AS-IV improved the spatial learning and memory deficits caused by chronic cerebral hypoperfusion. In addition, AS-IV reduced neuronal apoptosis, levels of oxidative damage mediators, and glial activation in the hippocampus. These results suggest that the ameliorating effect of AS-IV on learning and memory deficits might be the result of suppressing neuronal apoptosis and oxidative damage in the hippocampus.

## 3. Experimental Section

### 3.1. Animals

Male Sprague-Dawley rats (280–300 g, Nara Biotechnology, Seoul, Korea) were used for this study. All animal protocols were approved by the Ethics Committee for the Care and Use of Laboratory Animals at Kyung Hee University. The animals were housed in plastic cages maintained at constant temperature (22 ± 2 °C) and humidity (55% ± 10%) with 12 h–12 h light-dark conditions. The animals were allowed free access to food and water before the experiment.

### 3.2. Materials

AS-IV (from *Astragalus membranaceus*; C_41_H_68_O_14_; Figure 1A) was purchased from Biopurify Phytochemicals (Chengdu, China). Mouse anti-Bax antibody was purchased from Santa Cruz Biotechnology (Santa Cruz, CA, USA). Rabbit anti-caspase-3 antibody was purchased from Bioworld Technology (Louis Park, MN, USA). Mouse anti-4-HNE, mouse anti-8-OHdG, and rabbit anti-glial fibrillary acidic protein (GFAP) antibodies were purchased from Abcam (Cambridge, UK). Rabbit anti-ionized calcium binding adaptor molecule 1 (Iba1) antibodies were purchased from Wako Pure Chemical Industries (Osaka, Japan). Mouse anti-actin antibody was purchased from Chemicon International (Temecula, CA, USA). The other chemicals and reagents used were of high quality and obtained from various commercial sources.

### 3.3. Experimental Groups and Drug Treatment

Rats were randomly divided into five groups. The normal group was allowed free access to food and water without any operation and treatment. Data from the normal group were not shown and were used for normalization of the other data. The control group underwent anesthesia and cervical skin incision without pBCAO. The pBCAO group was subjected to pBCAO as described in the method section and was only administered the drug vehicle, which was an equal volume of normal saline. The AS-IV treatment group was treated with 10 mg/kg [AS-IV(10)] or 20 mg/kg [AS-IV(20)] of AS-IV (dissolved in 5% ethanol) delivered by intraperitoneal (i.p.) injection once a day for 28 days starting from four weeks after pBCAO. A total of 60 rats (12 rats per group) were used for this study. In pharmacokinetic study after intravenous injection of AS-IV to rats, brain tissue extracts contained ~0.1 µg/g tissue of AS-IV at dose of 1.5 mg/kg intravenous injection [38]. It is a small amount, but AS-IV obviously passes through the blood-brain barrier. The doses chosen for AS-IV administration were based on previous studies that provided neuroprotective effects in rat cerebral ischemia [14,16].

### 3.4. Permanent Bilateral Common Carotid Artery Occlusion

Chronic cerebral hypoperfusion in the rat brain was induced by pBCAO. Briefly, rats were anesthetized by i.p. injection of 18 mg/kg tiletamine and zolazepam (50:50, Zoletil, Virbac Laboratoris, Carros, France). The skin was incised along the midline of the cervical region, and the common carotid artery was dissected out without nerve damage. Both common carotid arteries were tied twice with silk suture. Afterwards, the skin incision was sutured and the animals were allowed to recover from the anesthesia on a warm pad. Animals that exhibited abnormal post-operative condition were excluded from the study.

### 3.5. Morris Water Maze Test

The Morris water maze test was performed for four days on the 9th week after pBCAO. The acquisition training was performed for three days and the retention test on the 4th day. The apparatus consisted of a circular water pool 190 cm in diameter and 40 cm in height. It was filled with 23 ± 1 °C water with a depth of 28 cm and covered a white platform (15 cm in diameter). The platform was submerged approximately 1 cm below the surface of the water. The pool was divided into four quadrants: northeast (NE), northwest (NW), southeast (SE), and southwest (SW) at equal distances from the rim. The platform was located in the center of the southwest quadrant. During the first 3 days acquisition test, the rats were given 8 trials per day to find the hidden platform. Each rat (12 rats per group) was gently placed into the water facing the wall in the direction of north (N), east (E), south (S), and west (W) in two series of order. The rat was allowed to swim until they reached the hidden platform (maximum swim time was 60 s). The escape latency to reach the platform was recorded and the rat was allowed to remain on the platform for 20 s before being removed. The rat which failed to find the platform within 60 s was guided to the hidden platform and then was placed on the platform for 20 s for reinforcement before being removed.

One trial of the retention test without the platform was performed on the 4th day to assess the rat’s memory of the correct platform location. The rats were placed into the pool and swam freely for 60 s. The swimming paths were recorded by a video camera linked to a computer-based image analyzer (SMART 2.5 video-tracking system, Panlab, Barcelona, Spain). The number of target heading and the swimming time in each zone was analyzed with a grid design of 6 zones (Figure 2A). This grid design, constructed with a computer-based image analyzer, was superimposed over the maze and viewed on a monitor. Memory scores were calculated using the formula [(time in zone A × 10) + (time in zone B × 8) + (time in zone C × 6) + (time in zone D × 3) + (time in zone F × 2) + (time in zone G × 1) = memory score]. The grid design and the formula for calculating the memory score were based on and modified from a previous behavior study [39]. The rats were sacrificed after the retention test trial and the brains were randomly used either for ELISA and western blotting (6 rats) or immunohistochemistry (6 rats).

### 3.6. Cresyl Violet Staining and TUNEL Labeling

Neuronal damage in the CA1 of the hippocampus was observed using cresyl violet staining and terminal transferase dUTP nick-end labeling (TUNEL) assay. The hippocampal tissue came from the rats which performed the Morris water maze test. The rats were deeply anesthetized and perfused transcardially with 0.05 M phosphate-buffered saline (PBS) containing 4% paraformaldehyde. The brain was removed and was postfixed in the same perfusing solution overnight at 4 °C. Thirty µm thick coronal sections of brain tissue were made using a freezing microtome (Leica, 2800N, Wetzler, Germany). TUNEL labeling was carried out using TACS 2 TdT-DAB in situ Apoptosis Detection kit (Trevigen, #4810-30-K, Gaithersburg, MD, USA) according to the manufacturer’s protocol.

### 3.7. Immunohistochemisty

The brain sections were stained by the free-floating DAB reaction. The sections were rinsed with 0.05 M PBS and incubated for 15 min in 1% hydrogen peroxide PBS at room temperature. The sections were incubated overnight at 4 °C with primary antibody against Bax (1:200, sc-7480, Santa Cruz Biotechnology), caspase-3 (1:500, BS1518, Bioworld Technology, Louis Park, MN, USA), 4-HNE (1:200, ab48506, Abcam,), 8-OHdG (1:500, ab62623, Abcam), GFAP (1:500, ab7260, Abcam), and Iba1 (1:500, #019-19741, Wako, Osaka, Japan), then incubated with biotinylated anti-mouse secondary antibody (1:200, Millipore, Billerica, MA, USA) for 1 h at room temperature, after which the avidin-biotin complex (Vector Laboratories, Burlingame, CA, USA) method was carried out with peroxidase coupling in a mixture containing 0.05% DAB (Sigma-Aldrich, St. Louis, MO, USA) and 0.03% H_2_O_2_ for 2–5 min. Images of the DAB-colorized brain sections were captured using a light microscope (BX51, Olympus, Tokyo, Japan) equipped with CCD camera (DP70, Olympus).

### 3.8. Image Analysis

Measurement of the CA1 neurons and thickness, TUNEL-labeled cells, relative optical densities of various immuno-labeled cells were analyzed using the ImageJ software (Ver. 1.44p, NIH, Bethesda, MD, USA). The number of CA1 neurons and TUNEL-labeled cells were counted in a 500 µm length of the pyramidal cell layer of the CA1. The relative optical densities were measured by the mean gray value on an inverted black-white binary image and were normalized to the value of the normal group. The percentage areas of astrocytes and microglia were measured on an inverted black-white binary image by determination of the threshold gray value and pixels definition using the ImageJ software. Data were normalized with the same area (10^5^ μm^2^) of the normal group. The mean values from the four sections analyzed in each rat were used for statistical analysis.

### 3.9. Measurement of SOD and MDA Levels

After the retention test trial, the cortical and hippocampal tissues were dissected separately and homogenized in Tris-HCl buffer. The homogenized samples were then centrifuged (3000 r/10 min) and the supernatant was collected for the various assays. The levels of SOD were measured by using the Superoxide Dismutase Activity Assay kit for xanthine oxidase system (STA-340, Cell Biolabs, San Diego, CA, USA). The levels of MDA were measured by using the MDA Adduct ELISA kit (STA-332, Cell Biolabs). All assays were done twice per sample, according to the recommendations of the manufacturer.

### 3.10. Western Blotting

The hippocampal tissue was homogenized and sonicated on ice in lysis buffer (50 mM Tris-HCl, pH 8.0, 150 mM NaCl, 1% Triton X-100, 0.5% sodium deoxycholate, 0.1% sodium dodecyl sulfate (SDS), 1 mM EDTA, 1% protease inhibitor cocktail; Sigma). After centrifugation, the supernatant was collected and assayed for protein concentration using the Bradford method. Lysate samples containing 50 µg of protein were fractionated by SDS-10% polyacrylamide gel electrophoresis, and then subjected to Western blot analysis. The primary antibodies used in this study were mouse anti-Bax antibody (sc-7480, Santa Cruz Biotechnology), rabbit anti-caspase-3 (D146, Bioworld Thecnology), rabbit anti-GFAP antibody (ab7260, Abcam), rabbit anti-Iba1 antibody (#016-20001, Wako), and mouse anti-β-actin antibody (Chemicon, Temecula, CA, USA).

### 3.11. ELISA

The contents of 4-HNE and 8-OHdG were measured in hippocampal tissue using specific ELISA kits (HNE Adduct Competitive ELISA Kit, STA-838; Oxidative DNA Damage ELISA Kit, STA-320; Cell Biolabs, San Diego, CA, USA) according to the manufacturers’ instructions.

### 3.12. Statistical Analysis

All data in this study are presented as means ± standard errors and evaluated using the Student’s *t*-test. A probability value of less than 0.05 was used to indicate a significant difference. Differences between the escape latencies between groups in the acquisition trials were evaluated using one-way ANOVA.

## 4. Conclusions

Oxidative damage in the brain plays a major role in the pathogenesis of neurodegenerative diseases including AD. Therefore, attenuation of oxidative damage is proposed as a valuable way to prevent dementia. The present study demonstrates that AS-IV ameliorates cognitive deficits via suppressing oxidative damage, neuronal apoptosis, and glial activation after chronic cerebral hypoperfusion. This suggests that AS-IV may be a potential therapeutic agent for dealing with AD and vascular dementia.
